# The reverse, but coordinated, roles of Tor2 (TORC1) and Tor1 (TORC2) kinases for growth, cell cycle and separase-mediated mitosis in *Schizosaccharomyces pombe*

**DOI:** 10.1098/rsob.110007

**Published:** 2011-11

**Authors:** Nobuyasu Ikai, Norihiko Nakazawa, Takeshi Hayashi, Mitsuhiro Yanagida

**Affiliations:** 1 Okinawa Institute of Science and Technology Promotion Corporation, 1919-1 Tancha, Onna, Okinawa 904-0412, Japan; 2 CREST Research Program, JST, Graduate School of Biostudies, Kyoto University, Sakyo-ku, Kyoto 606-8501, Japan

**Keywords:** target of rapamycin, rapamycin, Fkh1, Cdc2, separase

## Abstract

Target of rapamycin complexes (TORCs), which are vital for nutrient utilization, contain a catalytic subunit with the phosphatidyl inositol kinase-related kinase (PIKK) motif. TORC1 is required for cell growth, while the functions of TORC2 are less well understood. We show here that the fission yeast *Schizosaccharomyces pombe* TORC2 has a cell cycle role through determining the proper timing of Cdc2 Tyr15 dephosphorylation and the cell size under limited glucose, whereas TORC1 restrains mitosis and opposes securin–separase, which are essential for chromosome segregation. These results were obtained using the previously isolated TORC1 mutant *tor2-L2048S* in the phosphatidyl inositol kinase (PIK) domain and a new TORC2 mutant *tor1-L2045D*, which harbours a mutation in the same site. While mutated TORC1 and TORC2 displayed diminished kinase activity and FKBP12/Fkh1-dependent rapamycin sensitivity, their phenotypes were nearly opposite in mitosis. Premature mitosis and the G2–M delay occurred in TORC1 and TORC2 mutants, respectively. Surprisingly, separase/cut1—securin/cut2 mutants were rescued by TORC1/*tor2-L2048S* mutation or rapamycin addition or even Fkh1 deletion, whereas these mutants showed synthetic defect with TORC2/*tor1-L2045D*. TORC1 and TORC2 coordinate growth, mitosis and cell size control, such as Wee1 and Cdc25 do for the entry into mitosis.

## 2. Introduction

Understanding the relationship between cell division (increase in cell number) and growth (increase in cell volume or mass) is important in biology. It is well established that cyclin-dependent protein kinase (CDK) is the main regulator of division, while the target of rapamycin (TOR) complex regulates cell growth [1,2]. TOR controls a diverse set of cellular functions implicated in growth in response to nutritional changes. Rapamycin is an immunosuppressant drug commonly used in organ transplantation [3]. It was originally isolated as an antifungal compound produced by a bacterium [4]. It displays antiproliferative properties, prolongs the life of model animals, and might be useful in the treatment of certain cancers. Rapamycin binds to FK-binding protein (FKBP12, a peptidyl-prolyl cis-trans isomerase), which inhibits the TOR kinase complex (TORC) through direct binding [5]. The mammalian TOR (mTOR) catalytic subunit is the sole target of rapamycin through FKBP12 in mammals. In contrast, the budding yeast *Saccharomyces cerevisiae* has two kinases, Tor1 and Tor2. Frp1 (an FKBP12 homologue), Tor1 and Tor2 participate in rapamycin toxicity [6,7].

*Saccharomyces cerevisiae* Tor1 and Tor2 are closely related; they mediate the control of many cellular events, such as transcriptional activation, protein translation, ribosome biogenesis, cell cycle, nutrient uptake, actin organization and autophagy [8–14]. They also regulate cell growth in response to nutrient availability. Tor1 and Tor2 are large proteins with 80 per cent overall similar amino acid sequences with each other, containing several functional domains. Among such domains, the C-terminal catalytic serine/threonine kinase domain of Tor1/2 contains a conserved lipid kinase motif, which places the proteins in the phosphatidyl inositol kinase-related kinase (PIKK) family [15]. Tor1 and Tor2 form two functionally distinct TOR complexes [12]. TORC1, which is responsible for many of the known functions of TOR (reviewed in [16,17]), consists of either Tor1 or Tor2, together with the Kog1, Lst8 and Tco89 subunits [12]. TORC1 is sensitive to rapamycin, while TORC2 is rapamycin-insensitive [5]. TORC2 helps regulate cell wall integrity, receptor endocytosis and actin cytoskeleton polarization during cell cycle progression [14,18]. The inactivation of Tor2 disrupts actin organization [19]. TORC2 contains only Tor2, together with Avo1, Avo2, Tsc11, Lst8, Bit61 and Slm2 [20]. Rapamycin causes cell cycle arrest in the early G1 phase in *S. cerevisiae* [8].

The fission yeast *Schizosaccharomyces pombe* is an excellent model organism for the study of cell cycle control, mitosis, DNA damage repair, chromatin dynamics and meiosis. Similar to *S. cerevisiae*, *S. pombe* has two TOR kinase genes, *tor1*^+^ and *tor2*^+^ [21–29]. However, the nomenclature of TOR kinases in *S. pombe* is unfortunate: *S. pombe* Tor2 is similar to *S. cerevisiae* Tor1, whereas *S. pombe* Tor1 is similar to *S. cerevisiae* Tor2. Accordingly, TORC1 and TORC2 in *S. pombe* contain distinct subunits Tor2 and Tor1, respectively.

To couple extracellular nutrient signals to cell growth, *S. pombe* TORC1 and TORC2 are controlled by the small GTPases Rheb1 [30] and Ryh1 [31], respectively. Wild-type *S. pombe* is insensitive to rapamycin and vegetatively increases cell number in the presence of rapamycin [32]. However, *S. pombe* becomes sensitive to rapamycin under conditions of starvation. Analyses of the *fkh1* deletion mutant (Fkh1 is similar to mammalian FKBP12) suggest that rapamycin exerts its effect on sexual development in *S. pombe* by inhibiting the function of Fkh1 [21].

**Figure 1.**
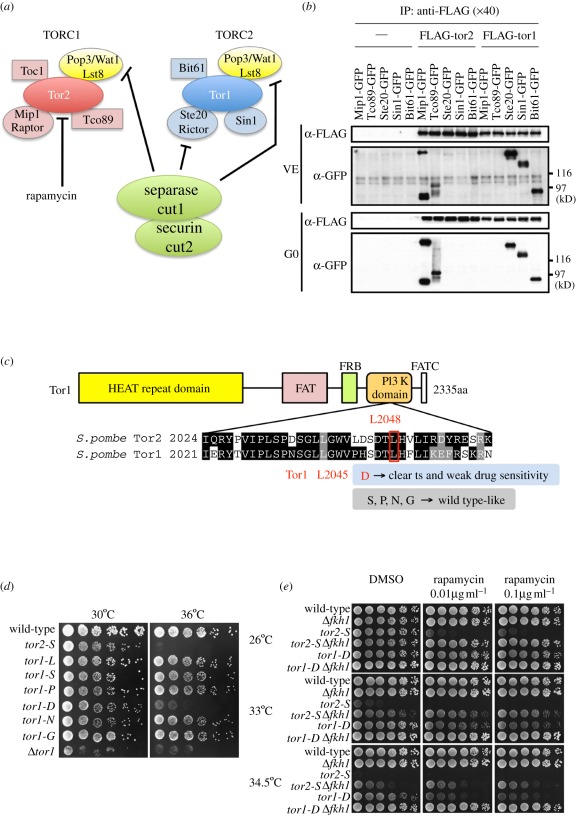
*Schizosaccharomyces pombe* TORC1 and TORC2 and construction of *tor1-D* mutant. (*a*) Schematic diagram of the subunit compositions previously determined by mass spectrometry [23]. The ts mutant *tor2-S* is highly sensitive to rapamycin. Multi-copy plasmid pCUT1 carrying the separase gene is inhibitory when introduced in *ste20-545*, *589* and *pop3/wat1-803* mutants [33] (Ste20 and Pop3/Wat1 are Rictor and Lst8 homologues, respectively). (*b*) Immunoprecipitation (IP) with anti-FLAG antibodies was performed for 15 strains that contained FLAG-tagged Tor2 or Tor1, and one of five GFP-tagged regulatory subunits (see text). A non-tagged strain (−) was used as negative control. Top: vegetatively (VE) growing cells were collected and immunoprecipitated by anti-FLAG antibodies. The resulting precipitates were run on SDS-PAGE and immunoblotted using anti-GFP antibodies. Bottom: the same experiment was performed, except that the cells were from the G0 phase. (*c*) Conserved amino acid sequences of *S. pombe* Tor2 and Tor1 PI3K domains are shown. L2048 of Tor2 corresponds to L2045 of Tor1. Five substitution (S, P, N, G and D) mutants at the L2045 residue were made. (*d*) Only the *L2045D* substitution produced the ts phenotype for *tor1* at 36°C, which is designated *tor1-D*. For controls, *tor2-L2048S* (previously constructed and designated *tor2-S* in the present study) and deletion Δ*tor1* are shown (see text). (*e*) The rapamycin sensitivity of *tor2-S* and *tor1-D* was examined in the presence of the deletion (Δ) of Fkh1 at various temperatures. See text.

Mass spectroscopic analysis of *S. pombe* TORC1 and TORC2 has revealed that each complex contains four evolutionarily conserved regulatory subunits, as schematized in figure 1*a* [23]. Mip1 and Ste20 [23,24,34] are homologues of mammalian Raptor and Rictor, respectively, while Pop3/Wat1 [33,35] is a homologue of Lst8 that associates with both TORC1 and TORC2. Using immunocoprecipitation with FLAG–Tor1 or FLAG–Tor2 (both chromosomally integrated and expressed under the control of the native promoter), Hayashi *et al.* [23] showed that Mip1, Pop3, Toc1 and Tco89 co-precipitate with FLAG–Tor2, while Sin1 [24,36], Pop3, Bit61 and Ste20 coprecipitate with FLAG–Tor1.

More recently, however, Hartmuth & Petersen [37] reported that the Tor1 catalytic subunit coprecipitates with Mip1 in minimal (synthetic) media, which suggests that *S. pombe* Tor1 and Tor2 may be the components of TORC1. To settle the apparently conflicting results, we reinvestigated in this study whether the TORC1-specific regulatory subunits Mip1 and Tco89 physically interact with both Tor1 and Tor2. Furthermore, for understanding distinct functions of Tor1 and Tor2, we constructed a conditional *tor1* mutant that allowed us to critically compare Tor1 functions with Tor2 functions in growth and cell cycle. In our previous study, the negative interaction was found between TORC mutants and overproduction of separase/Cut1 that is essential for mitosis [33]. Cut1 is a conserved protease that is essential for anaphase progression [38]. *Schizosaccharomyces pombe* Cut2 is a chaperon-inhibitor for Cut1 [39,40] and is degraded in the transition from metaphase to anaphase by the anaphase-promoting complex (APC)/cyclosome [41,42]. We wanted to clarify this unexpected link between TORC and mitosis. We will provide evidence that TORC1 and TORC2 are deeply implicated in cell division cycle control.

## 3. Results

### 3.1. TORC1 and TORC2 contain single, distinct catalytic subunits

To determine whether Tor1 is associated with only TORC2, immunoprecipitations were done. Ten strains containing chromosomally and doubly integrated FLAG-tagged Tor1 or Tor2 with one of the five green fluorescent protein (GFP)-tagged regulatory subunit genes (TORC1: Mip1 and Tco89; TORC2: Ste20, Sin1 and Bit61) were made (figure 1*b*). All of the tagged genes were expressed from their native promoters. As a control, five non-FLAG-tagged strains were also used for immunoprecipitation. All the GFP- and FLAG-tagged genes in this experiment were functional because every strain was viable, forming normal colonies and not sterile ones. These strains were vegetatively (VE) grown in the synthetic Edinburgh minimal medium 2 (EMM2) medium for 24 h at 26°C, and extracts immunoprecipitated with FLAG antibodies were subjected to immunoblot analysis using anti-GFP antibodies.

GFP-tagged Ste20, Sin1 and Bit61 coprecipitated abundantly with Tor1–FLAG, but GFP-tagged Mip1 or Tco89 did not. Conversely, GFP-tagged Mip1 and Tco89 coprecipitated abundantly with Tor2–FLAG, but GFP-tagged Ste20, Sin1 or Bit61 did not. Basically the same results were obtained in a similar experiment performed with G0 phase cells grown for 24 h at 26°C under conditions of nitrogen starvation (figure 1*b*, bottom). Longer exposures did not reveal any association between Tor1–FLAG and Mip1–GFP, nor between Tor1–FLAG and Tco89–GFP. These results demonstrated that the only detectable catalytic subunits of *S. pombe* TORC1 and TORC2 were Tor2 and Tor1, respectively. This is different from the case of budding yeast TORC1, which contains Tor1 and Tor2 [12]. In the quiescent G0 phase, the protein levels of TORC1 and TORC2 are roughly equal to those in the vegetative phase.

### 3.2. The substitution mutant *tor1-L2045D* is temperature-sensitive and fertile

In an effort to critically compare the mutant phenotypes of TORC1 and TORC2, we attempted to construct a conditional *tor1* substitution mutant. For this end, we used information about the mutation site of the previously isolated temperature-sensitive (ts) TORC1 *tor2-287* that resides in the highly conserved PI3 kinase domain and contains the substitution L2048S [23]. A comparison of the amino acid sequences indicated that Tor2L2048 corresponds to Tor1L2045 (figure 1*c*). Site-directed mutagenesis was performed at the Tor1L2045 site; however, the initial substitution mutant *tor1-L2045S* did not show any defect in colony formation at 26–36°C.

Several chromosomal integrants were then made by introducing amino acids P, N, G or D at the 2045 site. The *tor1-L2045D* mutant (designated *tor1-D* hereafter) showed the clear Ts^−^ phenotype, while the other mutants (L2045P, L2045N and L2045G) were Ts^+^ (figure 1*d*). The deletion mutant Δ*tor1* was previously isolated and characterized [43,44]. A comparison of the colony formations between Δ*tor1* and *tor1-D* showed that Δ*tor1* grew slowly, and was weakly ts and sterile. In contrast, *tor1-D* showed a clear ts phenotype at 36°C, but grew normally at 26°C and was fertile, which allowed the isolation of multiple mutants containing *tor1-D* by performing crosses at 26°C.

### 3.3. Fkh1 affects Tor1 and Tor2 in the presence or absence of rapamycin

As reported previously, *tor2-L2048S* (designated *tor2-S* hereafter) is highly sensitive to rapamycin (0.01 µg ml^−1^) at 26°C [23]. Thus, the next step in this study was to examine whether this sensitivity required the presence of Fkh1, an FKBP12-like protein that controls protein folding [45,46]. As expected from the presumed role of Fkh1 in generating rapamycin sensitivity in other organisms, the rapamycin sensitivity (0.01–1.0 µg ml^−1^) at 26°C was abolished in the double mutant *tor2-S* Δ*fkh1* (figure 1*e*, top row). In contrast, the *tor1-D* mutant was not sensitive to rapamycin at 26°C, but became significantly sensitive to the drug at the semi-permissive temperature of 33–34.5°C (middle and bottom rows, middle and right columns). In addition, the *tor1-D* mutant lost sensitivity at the semi-permissive temperature, when Fkh1 was deleted. Thus, the *tor1-D* mutation in the PIKK domain rendered the strain considerably sensitive to rapamycin at the semi-permissive temperature. The leucine uptake controlled by Tor1 was also sensitive to rapamycin in Fkh1-dependent manner [47]. Unexpectedly, the deletion Δ*fkh1* partly suppressed the ts phenotype of *tor2-S* at 33–34.5°C in the absence of rapamycin (dimethyl sulphoxide, DMSO). This finding will be discussed later, together with the finding that Δ*fkh1* suppressed other mutations.

### 3.4. Mutant target of rapamycin complexes contain the reduced kinase activities

**Figure 2.**
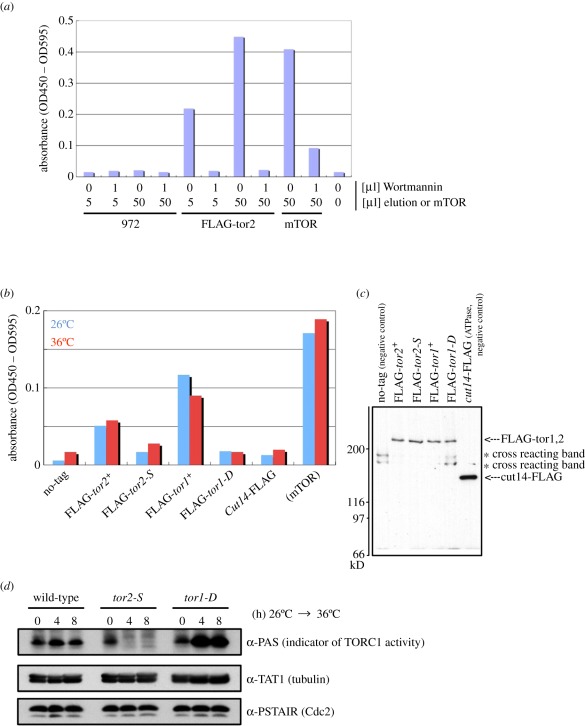
Diminished activities of mutant TORC1 and TORC2 and their cross talk interaction. (*a*) The kinase assay kit (K-LISA mTOR, Merck) was used with the human recombinant p70S6K-GST fusion protein as the substrate. The reaction mixture was incubated at 30°C, and the degree of phosphorylated S6KT389 was assayed by ELISA method. IP was conducted for the two strains, non-tagged 972 and chromosomally integrated FLAG-tagged tor2^+^. Immunoprecipitates from FLAG-tor2^+^ contained the dosage-dependent and Wortmannin-sensitive kinase activity, similar to mTOR. Extract 5 µl was equivalent to the IP obtained from 7 × 10^8^ cells. See text. (*b*) FLAG-tagged *tor1*^+^, *tor2*^+^, mutants *tor1-D* and *tor2-S* were employed for IP, and the TOR kinase activities of IP were measured. A positive control (mTOR) and negative controls (non-tagged and Cut14-FLAG) were also used. (*c*) Protein levels in the immunoprecipitates of FLAG-tagged proteins were assayed by immunoblot using anti-FLAG antibodies. The tagged strains used are all chromosomally integrated and expressed under the native promoters. (*d*) Immunoblot patterns of extracts of wild-type, *tor2-S* and *tor1-D* cultures grown at 26°C and then shifted to 36°C for 8 h. Antibodies against PAS (indicator of the TORC1 activity) and tubulin (TAT1) were used.

To understand the nature of ts *tor1-D* and *tor2-S* mutations, the kinase activities of immunoprecipitated FLAG-tagged Tor were measured using the authentic human substrate, the recombinant p70S6K-GST fusion protein, of which T389 was phosphorylated (purchased from Merck, Whitehouse Station, NJ). The non-tagged wild-type (972) precipitate showed no activity (figure 2*a*). In contrast, both mammalian and *S. pombe* TORC1, corresponding to purified mTOR (Merck) and immunoprecipitated FLAG–Tor2 (chromosomally integrated and expressed under the native promoter), respectively, displayed the activities, which were inhibited by the addition of Wortmannin (a known pharmacological inhibitor drug of PIK and PIKK [48]).

Next, the kinase activity levels of wild-type and mutant TORC1 and TORC2 were compared (figure 2*b*). The protein levels of immunoprecipitated FLAG–Tor2, FLAG–Tor2-S, FLAG–Tor1 and FLAG–Tor1-D were adjusted to roughly equal (figure 2*c*). Non-tagged and FLAG-tagged Cut14, a condensin SMC subunit, served as negative controls, and mTOR served as a positive control. The activity of the TORC1 mutant precipitated by FLAG–Tor2-S was diminished, and the activity of the TORC2 mutant precipitated by FLAG–Tor1-D was close to the background level at both 26°C and 36°C, suggesting that the mutations strongly reduced the kinase activities *in vitro*, but that the activities were not heat-labile.

### 3.5. TORC1 kinase activity might be upregulated in TORC2 *tor1-D* mutant cells

To obtain an insight into the *in vivo* activity of TORC1 in wild-type and mutant cells, antibodies against phospho-Akt substrate (PAS) were used for immunoblot to assay TORC1 activity [49]. These antibodies detected phosphorylated ribosomal protein S6, the presumed downstream substrate of TORC1. Three strains (wild-type, *tor2-S* and *tor1-D*) were cultured at 26°C, and then shifted to 36°C for 0–8 h. Extracts were prepared for immunoblot analysis, using antibodies against PAS, *α*-tubulin (TAT1) and Cdc2 (PSTAIR), the latter two of which were used as the loading controls. In *tor2-S* cells, the level of phosphorylated S6 greatly diminished after 4 h, which is consistent with the prediction that TORC1 activity was abolished in the mutant cells at 36°C (figure 2*d*). However, in *tor1-D* mutant cells, the degree of phosphorylated S6 sharply increased, which suggested that the activity of TORC1 (containing the wild-type Tor2) increased. This activation suggests that TORC1 activity might be strongly upregulated in the TORC2 mutant (*tor1-D*).

### 3.6. Mutants *tor1-D* and *tor2-S* have distinct phenotypes

**Figure 3.**
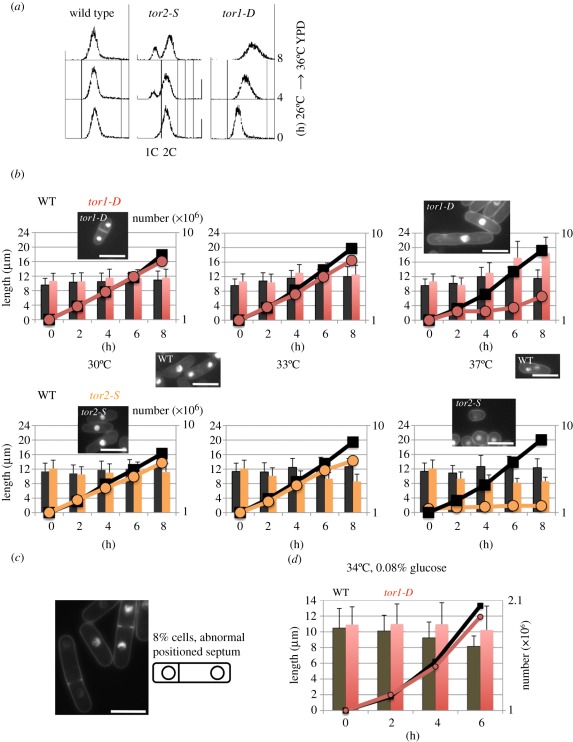
Phenotypic differences between *tor1-D* and *tor2-S*. (*a*) Wild-type, *tor2-S* and *tor1-D* initially grown at 26°C in YPD medium were shifted to 36°C for 0–8 h followed by measurement of DNA contents by FACScan. *tor1-D* shows the 2C peak that is shifted owing to the increase in cell length. (*b*) The cell length (micrometres, columns) and the cell number (line) of wild-type (black), *tor1-D* (red) and *tor2-S* (yellow) were measured for cells cultured initially at 26°C and then shifted to 30°C, 33°C and 37°C for 0–8 h. DAPI-stained cells taken at 8 h are also shown. (*c*) A small population of cells shows abnormally positioned septum. DAPI-stained *tor1-D* cells at 8 h. (*d*) Wild-type (*tor1*^+^) and *tor1-D* mutant were initially grown at 26°C in 2% glucose regular medium and then shifted to 34°C in low-glucose medium (0.08%). The cell number (line) and length (column, micrometre) are shown for wild-type (black) and *tor1-D* (red). (*b*,*c*) Scale bars, 10 µm.

Although the mutated residues in the PIK domain are the same, the phenotypes of *tor1-D* and *tor2-S* were dramatically different. FACScan (fluorescence-activated cell sorter scan; Becton Dickinson, Franklin Lakes, NJ) revealed that *tor1-D* cultured at 36°C for 0, 4 or 8 h displayed post-replicative 2C DNA content, while a pre-replicative 1C DNA peak was produced in *tor2-S* mutant at 36°C (figure 3*a*) [23]. Note that vegetatively growing *S. pombe* cells contain 2C DNA: pre-replicative nuclei exist in binucleated cells. The rightward shift of the FACS data does not reflect an increase in DNA content, but rather an increase in cell volume.

Changes in the cell number and cell length of *tor1-D* and *tor2-S* were then measured in the rich yeast extract, polypeptone, d-glucose (YPD) medium. The mutants were pre-cultured at 26°C, and then shifted to 30°C (permissive), 33°C (semi-permissive) or 37°C (restrictive temperature). The results are shown in figure 3*b*. At 30°C, *tor1-D* and *tor2-S* (red and orange lines) showed 4.7- and 3.7-fold increases in cell number, respectively, similar to the values for wild-type (black line). At 37°C, the cell number increase of both mutants ceased. Increasing the temperature to 37°C significantly increased the cell length of *tor1-D* (red column) by 60 per cent (inset, micrograph), but oppositely decreased that of *tor2-S* (orange column) by 33 per cent. After 8 h at 37°C, the average cell lengths were quite distinct: 18.5 ± 4.4 and 8.6 ± 1.2 µm for *tor1-D* and *tor2-S*, respectively. Those of the wild-type control for *tor1*^+^ and *tor2*^+^ were 11.5 ± 2.3 and 12.4 ± 2.5 µm, respectively. At 33°C, a semi-permissive temperature, the cell length of *tor2-S* significantly decreased, while the cell number increased. The cell length of *tor1-D* was the same as the wild-type.

These results suggested that cell division occurred prematurely for *tor2-S* but delayed for *tor1-D*, producing *wee1*-like and semi-*cdc25* phenotypes, respectively, at 37°C. Cells with the Δ*tor1* deletion were also further elongated [44]. Cells of *tor1-D* occasionally (8%) showed a misplaced septum (figure 3*c*). The phenotypes described here are consistent with the previous reports [24,25,28].

### 3.7. Cell-length shortening under low glucose does not occur in the *tor1-D*

*Schizosaccharomyces pombe* wild-type cells reduce in length in low-glucose medium (0.08% glucose), while they divide with the same doubling time as that in the regular medium (2% glucose) [50]. A decrease in glucose concentration from 2 to 0.08 per cent reduced the cell length of wild-type *tor1*^+^ strain (black column) by approximately 20 per cent after 6 h (figure 3*d*). In contrast, this cell-length shortening did not occur for *tor1-D* mutant (red column), which divided and increased the cell number (red line) at a similar rate to the wild-type (black line). Previously, we examined a number of ts and deletion mutants, and found that those mutants that failed to reduce cell length under low glucose were rare [51]. The *tor1-D* mutant shown here and *ssp1* mutants shown by Hanyu *et al*. [51] fail to reduce cell length under 0.08 per cent glucose.

### 3.8. Mitosis and cell division are delayed in *tor1-D*

**Figure 4.**
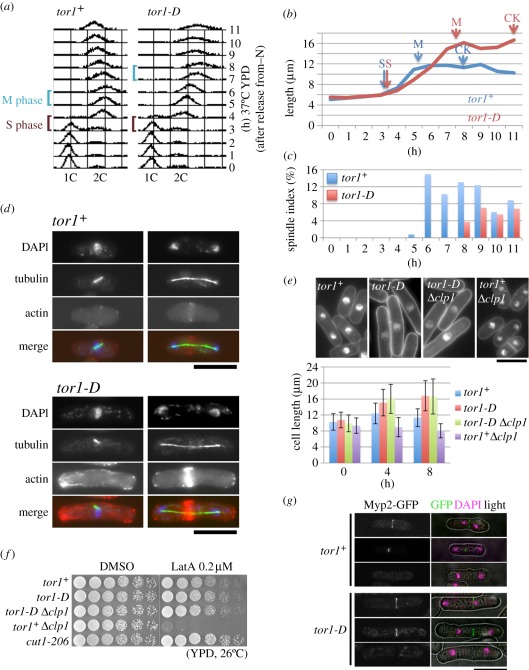
Mitotic delay and actin abnormality of *tor1-D*. (*a–c*) Wild-type (*tor1*^+^) and mutant *tor1-D* were brought into G0 phase by nitrogen starvation (EMM2-N) at 26°C for 24 h, and then released into nitrogen-replenished rich YPD medium at 37°C for 11 h. (*a*) The DNA content of cells after release from G0 was examined every 1 h by FACScan. S and M phase are indicated. (*b*) The cell length was measured after nitrogen replenishment at 37°C. Wild-type (blue line) and *tor1-D* (red line) are shown. (*c*) Immunofluorescence microscopy was performed to observe the mitotic spindle index using antibodies against tubulin (TAT1). (*d*) Micrographs of wild-type (*tor1*^+^) and mutant *tor1-D* cells stained by anti-tubulin, DNA-specific DAPI and actin-bound phalloidin. (*e*) The double mutant *tor1-D* Δ*clp1* was made, and cell length was compared with Δ*clp1* and *tor1-D*. See text. (*f*) Latrunculin A sensitivity was assayed for the single Δ*clp1* and the double mutant *tor1-D* Δ*clp1*. (*g*) The myosin ring was visualized by the chomosomally integrated myp2-GFP in the wild-type (*tor1*^+^) and mutant *tor1-D* cells. (*d*,*e*,*g*) Scale bars, 10 µm.

At 36°C, *tor2-S* was unable to grow in the nutrient-rich medium owing to its inability to properly use nutrients. As a result, the cells become short and round after one or two rounds of cell division (a few rounds of division are allowed in growth-deficient cells). The *tor2-S* mutant failed to exit from the G0 phase at 36°C, and remained small and round after the nutrient was replenished and cells were cultured at 36°C [23]. To examine whether *tor1-D* could properly exit from G0 phase, G0 cells of *tor1-D* were made under conditions of nitrogen starvation at 26°C for 24 h. Resulting G0 cells that looked normally round and small were harvested and transferred to rich YPD medium at 37°C. As shown in the FACS patterns (figure 4*a*), DNA replication (S phase) for the *tor1-D* mutant occurred at around 3–4 h after transfer to the YPD medium at 37°C, which was the same timing observed for wild-type G0 cells to return to S phase upon nutritional replenishment. However, 4′,6-diamidino-2-phenylindole (DAPI) staining of chromosomal DNA indicated that the first mitosis occurred about 7–8 h for the *tor1-D* mutant, showing considerable delay compared with wild-type *tor1*^+^ (figure 4*b*).

The frequencies of mitotic binucleated and septated cells, together with cell length, were measured for the wild-type and *tor1-D*. The average timings for S phase, M phase and cytokinesis (CK) are indicated by the arrows in figure 4*a*,*b*. While the timing of S phase was identical between wild-type and *tor1-D*, the timings of mitosis and CK were delayed by 2 h in *tor1-D* (red line). The cell length in *tor1-D* reached a plateau, at which point the cells were 30 per cent longer than the wild-type. The viability of *tor1-D* remained high (approx. 100%) at 37°C, while cell division was arrested. Taken together, *tor1-D* could exit from G0 and enter S phase with normal timing, but the G2–M transition was delayed while apparent cell growth continued (cell length increased).

### 3.9. The *tor1-D* mutant displays delayed spindle and aberrantly bright actin structures

The mitotic spindle in *tor1-D* was observed with an anti-tubulin antibody (TAT1), and the frequencies of cells showing the spindle were measured (red, figure 4*c*). The appearance of the spindle was 2–3 h delayed compared with wild-type. Figure 4*d* shows the short and long spindle by anti-tubulin, DNA by DAPI and actin by phalloidin in wild-type (top) and *tor1-D* (bottom), respectively. Unlike staining in the wild-type, mutant *tor1-D* displayed highly bright actin stain at one cell tip in interphase and at the equator in mitosis. In interphase, intense actin localization was seen at only one of the two tips in all of the cells examined, strongly suggesting that the new end failed to grow in *tor1-D*. In the wild-type, actin localization was bipolar after new end take-off (NETO) [52]. In electronic supplementary material, figure S1, more micrographs showing bright actin distributions at monopolar cell ends in interphase and at mitotic equator in *tor1-D* mutant are shown. Almost 100 per cent of *tor1-D* cells show the actin abnormality phenotype. The *tor1-D* mutant thus displayed the aberrant and asymmetric accumulation of actin at the ends of interphase cells.

### 3.10. *Tor1-D* is resistant to latrunculin A and overrides the deletion of Clp1/Flp1

In order to determine whether the delay in spindle formation was due to the activation of the CK checkpoint, which requires Clp1/Flp1 (similar to Cdc14 phosphatase) [53,54], the double mutant strain *tor1-D* Δ*clp1* was constructed (figure 4*e*; electronic supplementary material, figure S2). While control single-mutant Δ*clp1* cells displayed decreased cell length compared with wild-type cells at 36°C, the double mutant cells showed elongated phenotype that was indistinguishable from the single-mutant *tor1-D* cells. Cell length measurements in wild-type, single *tor1-D, double tor1-D* Δ*clp1* and single Δ*clp1* at 36°C suggested that the delayed CK in *tor1-D* was not dependent on Clp1/Flp1.

Sensitivity to the actin polymerization inhibitor latrunculin A was subsequently tested. As shown in figure 4*f*, the control Δ*clp1* mutant strain was sensitive to latrunculin A, while the double *tor1-D* Δ*clp1* mutant strain and the single *tor1-D* mutant strain were not. The control separase ts mutant strain *cut1-206* showed mild resistance at 26°C. We further examined the latrunculin sensitivity of *tor1-D* and *tor2-S* at the semi-permissive temperature (electronic supplementary material, figure S3). Both mutants were found to be considerably resistant to latrunculin at the permissive and/or semi-permissive temperature. Interestingly, the drug partly rescued *tor2-S* at 33°C. The double mutant *tor1-D* Δ*clp1* produced colonies nearly normally at 36°C in the presence of latrunculin. Considering that actin polymerization is inhibited by the drug [55], *tor1-D* and *tor2-S* might contain excessively polymerized actin that might inhibit colony formation. This finding is consistent with the cytological phenotype of *tor1-D*.

In order to determine whether the contractile ring in *tor1-D* cells was normal, we constructed wild-type and *tor1-D* strains in which chromosomally integrated and GFP-tagged Myp2/Myo3, the heavy chain of the myosin II complex [56], were expressed under the native promoter. As shown in figure 4*g*, GFP-tagged Myp2 in *tor1-D* at 37°C for 8 h was not intensely fluoresced like actin, and showed the normal-looking contractile ring during the period of CK. Taken together, while actin was abundant in *tor1-D* during interphase and mitosis, the defect was not found in the CK checkpoint nor in the contractile ring during mitosis. The above results are the basis for the further characterizations of mutant strains.

### 3.11. Cdc2 activation for mitosis may be delayed in *tor1*-*D* after the release from the G0 phase

**Figure 5.**
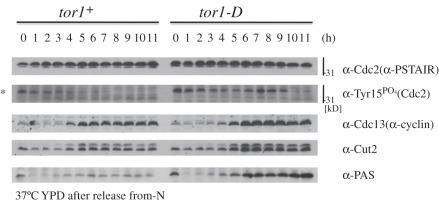
Dephosphorylation of Cdc2 Tyr15 PO_4_ is delayed in *tor1-D* mutant cells. The wild-type (*tor1*^+^) and *tor1-D* mutant were first nitrogen-starved at 26°C for 24 h in the EMM2-N medium, and then shifted to the replenished rich YPD medium at 37°C. The experimental design is identical to that in figure 4*a*–*c*. Aliquots of the culture media were taken at the 1 h intervals and extracts were run for immunoblot. Antibodies used were against Cdc2 (PSTAIR), Cdc2 (Tyr15PO_4_), Cdc13 (cyclin), Cut2 and PAS. The asterisk indicates the band position of phosphorylated Cdc2 at Tyr15. See text.

To determine whether the G2–M delay observed in *tor1-D* was due to the delay of Cdc2 activation, we employed antibody against Tyr15 PO_4_ (Cdc2) (a gift from T. Hunt [57]) to monitor phosphorylated Tyr15 residue of Cdc2 after the release from the nitrogen-starved G0 phase (figure 5). For other controls, antibodies against Cdc2 (PSTAIR), cyclin Cdc13, securin Cut2 and PAS are also shown.

Cdc2 Tyr15 was phosphorylated in the G0 phase of wild-type cells (the band indicated by the asterisk), consistent with a notion that the G0 phase contains the inactive Cdc2 as well as abundant Rum1, a Cdc2 inhibitor [58,59]. The Cdc2 Tyr15 PO_4_ band decreased around 3–5 h at the timing of the S to M phase (figure 4*a*,*b*; the degree of synchrony is not high). This timing roughly coincided with that of the disappearance of abundant Rum1 and the appearance of S phase cyclin [58]. In *tor1-D* mutant cells, the timing of the decrease of Cdc2 Tyr15 PO_4_ was greatly delayed until 9–11 h, roughly coinciding with the timing of M and cell division. Wu & Russell [59] showed that Cdc2 Tyr15 phosphorylation occurs under nitrogen starvation though the level of Cdc13 decreases. Considering the presence of Wee1 function under nitrogen starvation as the fact, it is surprising that premature mitosis can twice occur in the presence of Wee1. We hence consider that unidentified kinase in addition to Cdc2 may be implicated in Y15 phosphorylation in the cell division cycle arrest under nitrogen starvation. Sty1 mitogen-activated protein kinase (MAPK) is a candidate kinase that causes the size-shortening cell division [26,60].

The intense increase of Cdc13 occurred in wild-type around the S–M phase. The band intensities of Cdc13, Cut2 and phosphorylated PAS started to increase about 4 h, at the timing of S phase in both wild-type and *tor1-D*. The band intensity of PAS was much higher in *tor1-D* than that in wild-type, which is consistent with the result shown in figure 2*d*. Note that PAS was present in the G0 phase, but decreased temporally after the release. Taken together, the *tor1-D* mutation caused a great delay in the dephosphorylation of Cdc2 Tyr15 PO_4_, presumably delaying the activation of mitotic Cdc2 kinase, but parameters such as the timing of the increase for cyclin Cdc13, Cut2 and PAS after the release were basically identical to those of the wild-type.

### 3.12. Rapamycin suppresses the *cut1* and *cut2* mutants

**Figure 6.**
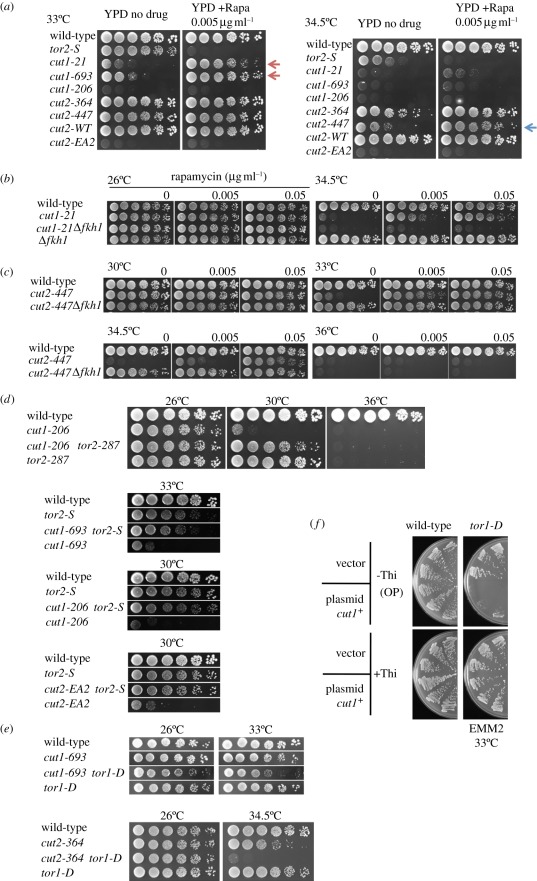
Rescue of ts *cut1* and *cut2* by rapamycin in the presence of Fkh1 or by *tor2*, and synthetic defects of *tor1* and *cut1* or *cut2*. (*a*) Rapamycin (0.005 µg ml^−1^) suppresses the ts phenotype of separase *cut1* and securin *cut2* mutants. Three *cut1* alleles (*-21*, *693* and *206*) and three *cut2* alleles (*-364*, *447* and *EA2*) were spotted on YPD plates and incubated in the absence or presence of rapamycin at 26°C, 30°C, 33°C or 34.5°C for 3–4 days. Wild-type and rapamycin-sensitive *tor2-S* are shown as controls. Results obtained at 26°C and 30°C are shown in electronic supplementary material, figure S4. (*b*) Effect of rapamycin on *cut1* mutants in the presence or absence of the Fkh1 gene. Δ*fkh1* is the deletion of Fkh1. (*c*) Effect of rapamycin on the *cut2-447* mutant in the presence or absence of the Fkh1 gene. (*d*) Synthetic rescue of *cut1* or *cut2* mutant by *tor2-S*. Two *cut1* and *cut2* mutants were used to construct the double mutants with *tor2-S*. The ts phenotypes of the resulting double mutants were examined. (*e*) Additive ts phenotype of the double mutants between *tor1-D* and *cut1-693* or *cut2-364*. (*f*) Synthetic inhibitory phenotype of plasmid pCUT1 and *tor1-D* mutant. See text.

As a negative genetic interaction existed between overproduced Cut1 and mutants of TORC2 regulatory subunits [33], the effect of rapamycin on the ts phenotype of the *cut1* and *cut2* mutants was tested. To our surprise, the ts phenotype of separase/*cut1* and securin/*cut2* mutants was rescued by rapamycin (0.005 µg ml^−1^). Rapamycin rescued *cut1-21* and *cut1-693* at 33°C, and *cut2-447* at 34.5°C, as shown in figure 6*a*. Rapamycin also suppressed the ts phenotype of *cut1-206* and *cut2-EA2* mutants (indicated by red and blue arrows, respectively) at 30°C, a restrictive temperature for these strains (electronic supplementary material, figure S4). These data show that all of the three *cut1* and two *cut2* ts alleles examined were suppressed by a very low concentration of rapamycin, suggesting that the rescue was highly effective and not allele-specific. The degree of suppression for *cut1* seemed to be stronger than for *cut2* mutants. The control strain *tor2-S* showed strong drug sensitivity at 33°C, a semi-permissive temperature. The hyper-sensitive and rescue effects by rapamycin show that Tor2 and securin–separase respond to rapamycin in opposing manners.

### 3.13. Fkh1 is deeply implicated in the suppression of *cut1* and *cut2* mutants

We then tested the effect of Fkh1 on rapamycin-mediated suppression of separase/*cut1* mutants. Three separase mutants, *cut1-206, -693* and *-21*, were crossed with Δ*fkh1*. Resulting double mutants were cultured in the absence or the presence of rapamycin (0.005 and 0.05 µg ml^−1^). The rescue by rapamycin for *cut1-21* and *cut1-693* at the semi-restrictive temperature was abolished in Δ*fkh1* (figure 6*b*; electronic supplementary, figure S5). For the case of *cut1-206* Δ*fkh1*, synthetic rescue already occurred at 30°C in the absence of rapamycin (electronic supplementary material, figure S5). This intriguing observation is discussed below.

Even stronger suppression was found for the double mutant *cut2-447* Δ*fkh1* in the absence of rapamycin at 33°C and 34.5°C (figure 6*c*). The suppression effect was further strengthened by the addition of rapamycin. This unexpected result suggested that Fkh1 acts on securin–separase in two ways, in the presence and the absence of rapamycin. Fkh1 may enhance the instability of Cut2 protein in the absence of rapamycin, and is required for the ts phenotype of the *cut2* mutant.

### 3.14. Separase and securin mutants are rescued by the TORC1 mutation *tor2-S*

We then tested a hypothesis that rapamycin-sensitive *tor2-S* mutation could substitute rapamycin addition in the above experiments. The double mutant *tor2 cut1* might represent a situation similar to single *cut1* mutant in the presence of rapamycin. Two double mutants of *cut1-206* or *cut1-693* were made with *tor2-287* (original ts isolate having the same mutation site to *tor2-S* [23]) and *tor2-S* (constructed by substitution through chromosome integration). The synthetic rescue at 30°C and 33°C was strong for all of the combinations of *cut1 tor2* double mutants (figure 6*d*). We also tested whether the same was true for the case of securin/*cut2* mutation. At 30°C, the synthetic rescue was clearly observed for the double *cut2 tor2* (*cut2-EA2 tor2-S*). Taken together, diminishing the catalytic subunit Tor2 of the TORC1 greatly alleviates the requirement of separase Cut1 and securin Cut2 in *S. pombe*. Thus, separase/Cut1–securin/Cut2 complex, the central player of chromosome segregation, was deeply implicated in the nutrient sensor TORC1 in a manner suggesting that their functions may be opposing.

### 3.15. Combination of *tor1-D* and *cut1* or *cut2* was additive

To test whether TORC2 mutation *tor1-D* genetically interacts with mutations of *cut1* or *cut2*, double mutants were made. The resulting ts phenotypes of *tor1-D cut1-693* and *tor1-D cut2-364* showed additive effects: at 33°C, the single mutants *cut1-693* and *tor1-D* formed colonies, but the double mutant showed slow formation of colonies (figure 6*e* top). The single mutants *cut2-364* and *tor1-D* produced colonies at 34.5°C, but the double mutant *tor1-D cut2-364* hardly formed colonies (figure 6*e*, bottom). A possible explanation for the additive phenotypes of *cut2* and *cut1* mutants with *tor1-D* is that TORC2 (Tor1) acts in parallel with the Cut1–Cut2 complex. Securin–separase and Tor1 might share an essential function.

Another additive effect found was between *tor1-D* and plasmid pCUT1, as shown in figure 6*f*. The colony formation of *tor1-D* at 33°C, a semi-permissive temperature, was strongly inhibited when Cut1, under the control of the inducible *nmt1* promoter on pCUT1, was overproduced in the absence of thiamine (−Thi). This result is consistent with the fact that overproduction of Cut1 was inhibitory to the colony formation of *ste20* [33]. Besides the presumed parallel function of TORC2 and Cut1–Cut2, the unbalanced high dosage of Cut1 seems to be harmful to the diminished situation of TORC2.

### 3.16. Rapamycin allows *cut1-206* to divide, but does not restore the protein level

**Figure 7.**
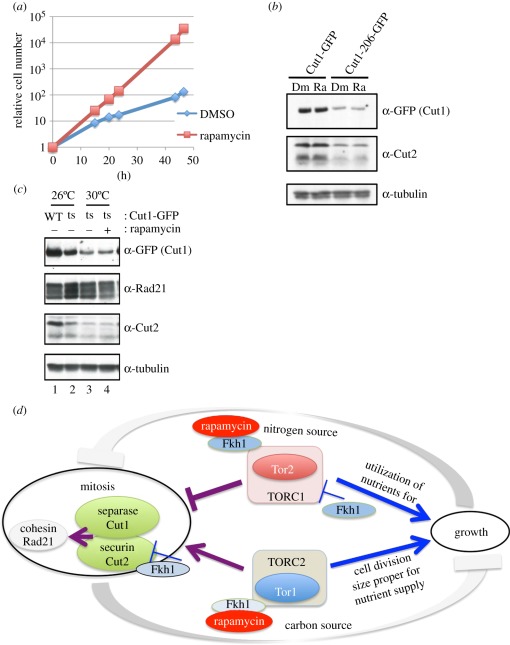
Securin and separase are scarce during rescue. (*a*) GFP was chromosomally tagged at the C-terminus of the *cut1-206* mutant gene. The resulting strain *cut1-206-GFP* was cultured at 30°C in the presence (rapamycin) or absence (DMSO) of rapamycin (4 µg ml^−1^), and the cell number increase was measured. (*b*) The wild-type strain carrying the *cut1*^+^ gene was chromosomally tagged with GFP at the C-terminus. The protein levels of the resulting Cut1-GFP and mutant Cut1-206-GFP proteins were estimated in the presence (4 µg ml^−1^, Ra) or absence (Dm) of rapamycin at 26°C by immunoblot using anti-GFP antibodies. For comparison, the immunoblot of Cut2 and tubulin (loading control) were performed using polyclonal anti-Cut2 and monoclonal anti-TAT1 antibodies, respectively. (*c*) Lanes 1 and 2 show wild-type Cut1–GFP and mutant Cut1-206-GFP, respectively, grown in the absence of rapamycin at 26°C. Lanes 3 and 4 show mutant *cut1-206-GFP* cultured at 30°C in the presence or absence of rapamycin (4 µg ml^−1^), respectively. Immunoblot was performed to estimate the levels of the wild-type and mutant Cut1 (GFP). The levels of Rad21, Cut2 and tubulin were also estimated. The addition of rapamycin does not change the level of mutant Cut1-206-GFP. (*d*) A speculative explanation for the distinct mutant phenotypes of *tor1-D* and *tor2-S* for the control of mitosis and growth, and the cartoon describing the relationship between TOR complexes and securin–separase. See §4.

To investigate how the *cut1-206* mutant was suppressed in the presence of rapamycin, the mutant strain that expressed *cut1-206-GFP* mutant protein under the native promoter was constructed and grown in the liquid culture in the presence or the absence of rapamycin (4 µg ml^−1^) at 30°C for 46 h. The mutant cells clearly divided more frequently in the presence of rapamycin than in its absence, with doubling times of 3.1 and 6.6 h, respectively (figure 7*a*). This result indicated that the suppression occurred at the level of doubling time, and also suggests that the level of the Cut1-206-GFP mutant protein might increase in the presence of rapamycin. To test this hypothesis, immunoblot was done for comparing the levels of mutant Cut1-206-GFP protein in the presence or absence of rapamycin. Contrary to the hypothesis, the level of Cut1-206-GFP protein did not increase at all in the presence of rapamycin. The level at 26°C was lower than that of the wild-type Cut1–GFP, regardless of the rapamycin status (figure 7*b*). The level of Cut2 protein was also low in the mutant cells.

The level of the Cut1-206-GFP protein was further examined under the suppressed condition in the presence of rapamycin at 30°C for 46 h (figure 7*c*). The levels of Cut1-206-GFP and Cut2 did not increase at 30°C regardless of the presence (+) or the absence (−) of rapamycin. Thus, the strong rescue of *cut1-206* by rapamycin did not accompany the increase for the level of Cut1 and Cut2. The level of Rad21, a cohesin subunit that was cleaved by activated separase/Cut1 [61], also did not change at 30°C in the suppressed (+ rapamycin) or non-suppressed (−drug) condition. Rapamycin suppressed the ts phenotype of *cut1* mutation not through the increase for the level of Cut1 and/or Cut2. We discuss below how diminishing TORC1 could suppress *cut1* and *cut2* mutations.

## 4. Discussion

We were able to critically compare the mutant phenotypes of *S. pombe* TORCs by constructing the new mutant *tor1-L2045D (tor1-D)*, a substitution at the same conserved residue as that of previously isolated *tor2-L2048S (tor2-S)*. Mutant TORCs purified from these mutant extracts showed diminished kinase activities, consistent with the mutation site in the PIK domain. However, the phenotypes of *tor1-D* and *tor2-S* were greatly distinct and nearly opposite in a number of phenotypes examined. The amino acid sequences of *S. pombe* Tor1 and Tor2 are similar throughout except for the amino terminal region that is unknown for the functional relevance. How did the similar mutations produce such different phenotypes? Stably bound regulatory subunits, such as Mip1 or Ste20, or other proteins like Fkh1, which might interact with the mutation sites, are probably crucial for producing such differences. As the single TOR catalytic subunit can produce both TORC1 and TORC2 in many organisms, including mammals (mTOR), it is of interest to introduce the same mutations of *tor1-D* and *tor2-S* into the genomic TOR, and examine their phenotypes. Phenotypes specific for TORCs are possibly produced.

The defective phenotypes of *S. pombe* TORCs have been intensively studied [22–24,27,28,47,62,63]. While the deletion mutant Δ*tor1* is viable, though sterile, and slow in colony formation [27,47], *tor1-D* single substitution mutant made in this study is normal in colony formation and cell size at 26°C, and fertile, but fails to form colonies at 37°C. The *tor2*^+^ gene is essential for viability, and the *tor2-S* mutant cells at 36°C resemble the quiescent wild-type cells produced under nitrogen source starvation, which induces two rounds of mitosis and cell division in the absence of cell growth [64]. By contrast, Tor1 (TORC2), Ste20, a TORC2 regulatory subunit and TORC2-related kinases such as Ksg1 and Gad8 are required for proliferation under low-glucose conditions (S. Saitoh & M. Yanagida 2011, unpublished data). TORC1 and TORC2 may hence be distinguished in response to different nutrients, such as amino acids and low glucose, respectively.

The emerging roles of TORC1 and TORC2 in cell size control are discussed below. On the one hand, it is well known that Wee1 and Cdc25 control the cell size of dividing *S. pombe* [65]. These are cell cycle regulators and control Cdc2 kinase, a master regulator of cell cycle, in opposing ways. Both Wee1 and Cdc25 affect the cell size via inhibiting and activating Cdc2 kinase, respectively, but are not required for cell growth, as *cdc25* and *wee1* mutant cells can increase in cell length. On the other hand, cell growth is also involved in the cell size control; nutrients such as nitrogen and carbon sources affect the cell size. Under nitrogen-source starvation as described above, *S. pombe* cells became small and round. Under low-glucose conditions, the size of dividing cell is shortened, probably to keep the short doubling time [50]. As shown in this and earlier studies, two growth-related kinases, TORC1 and TORC2, are clearly implicated in cell size control, and their apparent functions in cell size control are opposite. At 36°C, *tor1-D* cells are elongated, while *tor2-S* cells are short. We postulate that Tor1 (TORC2) and Tor2 (TORC1) coordinate to determine the cell size in response to nutritional conditions, and resemble Cdc25 and Wee1, respectively, regarding the positive and negative roles for cell division under nutritional supply. A key observation is that under low glucose concentration, the wild-type cell length is shortened, but the length of dividing *tor1-D* cells is elongated. In other word, *tor1-D* mutant cells fail to reduce cell length in response to limited glucose concentrations. This failure may be related to aberrantly intense and monopolar actin localization in interphase cells. We further showed that the delay phenotype of *tor1-D* is not due to CK checkpoint, but due to the delay of dephosphorylation timing of Cdc2 Tyr15 PO_4_ for the mitotic entry. Negative and positive genetic interactions between mutants of TORC2 and *cdc25* or *wee1*, respectively, have been reported [62,63]. Tor1 may be required for Cdc2 kinase activation via direct or indirect activation of Cdc25 or through inhibition of Wee1.

The TORC2 mutant *tor1-D* is moderately sensitive to rapamycin at the semi-permissive temperature, while *tor2-S* is highly sensitive at 26°C. The sensitivity requires the presence of FKBP12-like Fkh1, as expected from previous studies [12,46,47]. In the absence of rapamycin, however, Fkh1 still affected Tor2: the ts phenotype of *tor2-S* at 33–34.5°C was suppressed by the deletion of Fkh1. The FKBP of higher eukaryotes does not interact with the rapamycin-interacting FRB domain in the absence of rapamycin [66]. *Schizosaccharomyces pombe* Fkh1 might influence the function of Tor2 via a domain other than FRB. Fkh1 may negatively regulate the stability of Tor2 mutant protein, such that the Tor2-S mutant protein is restored in the absence of Fkh1.

Strong functional implication of Fkh1 to mitosis was obtained. The ts phenotype of securin/*cut2* and separase/*cut1* was rescued by the deletion of Fkh1 in the absence of rapamycin. The rescue was particularly strong for *cut2* mutant. The mechanism for this rescue is unclear, but protein conformation of Cut2 seems to be strongly affected by Fkh1 in the absence of rapamycin. Hence Fkh1, a protein-folding accelerator, is required for the ts *cut2* phenotype through destabilizing mutant Cut2 protein. Note that mammalian securin is non-essential, though intense chromosome instability leading to cancer can be caused by overproduction. Conformation-sensitive phenotypes of *S. pombe cut1* and *cut2* mutants were previously reported [67]; high concentrations of osmotic compound (1.2 M sorbitol) or salt (0.6 M KCl) rescued *cut1* and *cut2* mutants. In this rescue, the level of mutant Cut1–Cut2 complex increased in a manner dependent on the actions of stress-responsive MAPK Sty1/Spc1. Rescue by rapamycin, however, did not accompany the protein level increase of Cut1 and Cut2, suggesting that only small amounts of Cut1 and Cut2 may be needed in rapamycin-treated cells.

How do we explain the synthetic rescue of securin/*cut2* and separase/*cut1* mutations by either rapamycin addition or *tor2-S* mutation? The balance between TORC1 and securin–separase appears to be crucial for avoiding premature mitosis and chromosome missegregation (figure 7*d*). There is a popular belief that growth opposes cell division. Similar to this concept, mitosis may be restrained, while Tor2 (TORC1) is active. Securin–separase may have to be intensely active, when Tor2 is abundant. Conversely, when the TORC1 activity of dividing cells is diminished, the requirement of securin–separase may be greatly alleviated. These considerations explain many results obtained in this study. TORC1 may prevent premature entry into mitosis, so that *tor2-S* or rapamycin-treated cells alter strikingly the mode of mitosis and cell division, leading to small cells even in the rich medium. Wild-type cells brought under nitrogen starvation actually commit premature mitosis with regard to cell size, which is consistent with the notion that TORC1 becomes inactive upon removal of nitrogen source (reviewed by Yanagida *et al*. [68]).

*Schizosaccharomyces pombe* may have abundant TORC1 activity in the regular vegetative culture medium, which may explain why rapamycin shows little effect on cell division of wild-type cells [32]. Under such conditions, the levels of Cut1–Cut2 might be abundant to balance against TORC1. When Tor2-mediated growth support is diminished in mutant cells, however, only a tiny amount of Cut1–Cut2 may be sufficient. Note that biologically important events such as immunological responses are suppressed in mammalian cells by rapamycin. In sharp contrast, Tor1 (TORC2) seems to act in parallel with mitosis through regulating the CDK activation and actin localization. Tor1 appears to have a forward role in the progression of mitosis because of its presumed function in determining the timing of mitosis. Tor1 and Cut1–Cut2 may coordinate chromosome segregation and cell division, thereby ensuring determination of appropriate cell size (short size in low glucose). In other organisms, such as *S. cerevisiae*, TORC2 is known to be related to actin distribution [11,14,20] and is thought to regulate the cell cycle-dependent polarization of the actin cytoskeleton. The initiation of bipolar cell elongation in *S. pombe* is actually highly complex: more than 30 protein kinases are implicated [52]. In TORC1 (*tor2*) mutant, overall actin organization is reported to be normal [27], but detailed study may be necessary as *tor2-S* mutant is resistant to latrunculin. In short, whereas TORC1 and TORC2 are apparently opposing, they should actually coordinate to support growth and determine the timing of division. Our previous results [69] showed that the phenotypes of *cut* (cell untimely torn) and *wee* (small) were both observed in the double mutant of *cut1* and *wee1*. The suppression of *cut1* by *tor2* should thus be really a specific phenotype of *tor2*. It remains to be determined whether Cut1 or Cut2 is the direct phosphorylation target of TORC1 and TORC2 kinases.

While *S. cerevisiae* TORC1 and TORC2 contain two and one catalytic subunits, respectively, the present study unequivocally demonstrated that *S. pombe* TORC1 and TORC2 each contained one catalytic subunit in vegetative and G0-quiescent cultures within the experimental resolution. The present result differs from that of Hartmuth & Petersen [37], but is consistent with that of Hayashi *et al*. [23]. The reason for the difference is unclear, but might be due to the use of Tor1-HA gene expressed under non-induced state of the highly inducible *nmt1* promoter by Hartmuth & Petersen, while our study relied on chromosomally integrated genes expressed under the native promoters. This issue is important: if TORC1 contained both Tor1 and Tor2 catalytic subunits, the interpretation of our results regarding distinct phenotypes of *tor1* and *tor2* mutations would be less straightforward. Additionally, we showed that, between proliferation and quiescence, *S. pombe* TORC1 and TORC2 constituents were the same, and their levels were similar. The role of plentiful TORC1 and TORC2 in quiescence may be storage for the restart of growth. However, the PAS activity was high in the G0 cells, so that TORC1 may give a high PAS activity in the G0 phase cells that do not grow at all. Alternatively, another kinase may be responsible for the PAS activity in the G0 cells.

## 5. Material and methods

### 5.1. Strains, materials and general techniques

*Schizosaccharomyces pombe* heterothallic haploids 972 *h*^−^, 975 *h*^+^ and their derivatives were used. Strains used in this study are listed in table 1. Complete YPD (1% yeast extract, 2% polypeptone and 2% glucose) and minimal EMM2 were used to culture *S. pombe*. The phenotype of *tor1-D* was affected by nutritional condition: it was somewhat unclear in the synthetic EMM2 medium, but clear in the YPD medium. The nitrogen-starved G0 phase cells were prepared as follows [71]. The cells were grown in EMM2 to a concentration of 5 × 10^6^ cells ml^−1^ at 26°C. They were harvested by vacuum filtration using a nitrocellulose membrane (0.45 µm pore size; Millipore, Billerica, MA), washed in EMM2–N (EMM2 lacking NH_4_Cl) once on the membrane, and then resuspended in EMM2–N at a concentration of 5 × 10^6^ cells ml^−1^ and incubated at 26°C for 24 h. The transient G1 cells formed after approximately two divisions during the first 4–6 h under nitrogen starvation became G0 cells after 12 h [64]. Nitrogen was replenished by adding fresh EMM2 medium to reach a concentration of 1 × 10^6^ cells ml^−1^. None of the strains used for nitrogen starvation was an auxotroph. FACScan analysis was performed as described previously [71]. For the culture of *S. pombe* in limited glucose [50], cells grown in EMM2 containing 2 per cent glucose were collected by centrifugation and washed in 0.08 per cent glucose medium, and cultured in EMM2 medium containing 0.08 per cent glucose. Rapamycin was obtained from Sigma-Aldrich (St Louis, MO). For immunoblot, anti-Tyr15 PO_4_ (Cdc2; a gift from Dr Tim Hunt), anti-TAT1 (a gift from Dr Keith Gull) and anti-FLAG M2 (Sigma) were used. Anti-PAS antibody (Cell Signaling Technology, Inc., Danvers, MA) was used for immunoblot as described previously [49]. The Δ*clp1* strain was provided by the Yeast Genetic Resource Centre (YGRC).

**Table 1.** *Schizosaccharomyces pombe* strains used in this study.

strain	genotype	source
NI1123	*h*^−^*mip1-GFP:hygR*	this study
NI1121	*h*^−^*tco89-GFP:natR*	this study
NI1124	*h*^−^*ste20-GFP:hygR*	this study
NI1125	*h*^−^*sin1-GFP:hygR*	this study
NI1122	*h*^−^*bit61-GFP:natR*	this study
NI1166	*h*^−^*FLAG-tor2:kanR mip1-GFP:hygR*	this study
NI1164	*h*^−^*FLAG-tor2:kanR tco89-GFP:natR*	this study
NI1167	*h*^−^*FLAG-tor2:kanR ste20-GFP:hygR*	this study
NI1168	*h*^−^*FLAG-tor2:kanR sin1-GFP:hygR*	this study
NI1165	*h*^−^*FLAG-tor2:kanR bit61-GFP:natR*	this study
NI1161	*h*^−^*FLAG-tor1:hygR mip1-GFP:hygR*	this study
NI1159	*h*^−^*FLAG-tor1:hygR tco89-GFP:natR*	this study
NI1162	*h*^−^*FLAG-tor1:hygR ste20-GFP:hygR*	this study
NI1163	*h*^−^*FLAG-tor1:hygR sin1-GFP:hygR*	this study
NI1160	*h*^−^*FLAG-tor1:hygR bit61-GFP:natR*	this study
NI0001	*h*^−^*972*	laboratory stock
NI0002	*h*^+^*975*	laboratory stock
NI993	*h*^−^*tor2-L2048S:kanR*	[23]
NI1079	*h*^−^*tor1-L2045L:hygR*	this study
NI1080	*h*^−^*tor1-L2045S:hygR*	this study
NI1081	*h*^−^*tor1-L2045P:hygR*	this study
NI1082	*h*^−^*tor1-L2045D:hygR*	this study
NI1083	*h*^−^*tor1-L2045N:hygR*	this study
NI1084	*h*^−^*tor1-L2045G:hygR*	this study
NI1048	*h*^−^*tor1Δ::hygR*	this study
NI1145	*h*^−^*fkh1Δ::natR*	this study
NI1215	*h*^−^*tor2-L2048S:kanR fkh1Δ::natR*	this study
NI1213	*h*^−^*tor1-L2045D:hygR fkh1Δ::natR*	this study
NI1046	*h*^−^*FLAG-tor2:kanR*	[23]
NI1261	*h*^−^*FLAG-tor2-287:kanR*	this study
NI1254	*h*^−^*FLAG-tor1-L2045L:hygRkanR*	this study
NI1255	*h*^−^*FLAG-tor1-L2045D:hygRkanR*	this study
NN0001	*h*^+^*ura4 cut14-3FLAG:ura4*^+^	this study
NI1212	*h*^−^*tor2-L2048L:kanR*	this study
NN0002	*h*^−^*tor1-L2045D:hygR clp1Δ::kanR*	this study
NN0003	*h*^−^*tor1-L2045L:hygR clp1Δ::kanR*	this study
NN0004	*h*^−^*cut1-206*	[41]
NN0005	*h*^+^*tor1-L2045L:hygR Myp2-GFP:kanR*	this study
NN0006	*h*^+^*tor1-L2045D:hygR Myp2-GFP:kanR*	this study
NI1022	*h*^+^*cut1-21*	[41]
NI1014	*h*^+^*cut1-693*	[41]
NI1012	*h*^+^*cut1-206*	[41]
NI1016	*h*^+^*cut2-364*	[41]
NI1026	*h*^−^*cut2-447*	[39]
NI1035	*h*^−^*ura4 cut2WT:ura4*^+^	[70]
NI1036	*h*^−^*ura4 cut2EA2:ura4*^+^	[70]
NI1403	*h*^−^*cut1-21 fkh1Δ::natR*	this study
NI1310	*h*^+^*cut2-447*	[39]
NI1336	*h*^+^*cut2-447 fkh1Δ::natR*	this study
NI1415	*h*^+^*cut1-206 tor2-287*	this study
NI1412	*h*^−^*tor2-287*	[23]
NI1013	*h*^+^*cut1-693 tor2-L2048S:kanR*	this study
NI1011	*h*^+^*cut1-206 tor2-L2048S:kanR*	this study
NI1050	*h*^−^*ura4 cut2WT:ura4*^+^*tor2-L2048S:kanR*	this study
NI1051	*h*^−^*ura4 cut2EA2:ura4*^+^*tor2-L2048S:kanR*	this study
NI1089	*h*^+^*tor1-L2045D:hygR*	this study
NI1103	*h*^+^*cut1-693 tor1-L2045D:hygR*	this study
NI1335	*h*^+^*cut2-364 tor1-L2045D:hygR*	this study
NI1316	*h*^−^*leu1 tor1-L2045D:hygR*	this study
NI1399	*h*^+^*cut1-GFP:kanR*	this study
NI1401	*h*^+^*cut1-206-GFP:kanR*	this study
NN0007	*h*^+^*cut14-208*	this study
NI1407	*h*^−^*cut1-693 fkh1Δ::natR*	this study
NI1405	*h*^−^*cut1-206 fkh1Δ::natR*	this study

### 5.2. Isolation of temperature-sensitive *tor1-D* strain

To construct various mutant strains substituted at the L2045 residue of Tor1 to L, S, P, D, N or G, the carboxy-terminal 2.3 kb of the *tor1*^+^ open reading frame was cloned into the pHYG- or pKAN-derivative plasmids (containing the antibiotic-resistant marker), which were PCR mutagenized for different substitutions. Chromosome integration onto the endogenous *tor1*^+^ locus was followed, and the resulting integrant strains were tested for ts phenotype. *tor1-D* was the only mutant showing the ts phenotype.

### 5.3. Examination of the association between target of rapamycin complex catalytic and regulatory subunits

The TORC catalytic and regulatory subunits were tagged by FLAG and GFP, respectively. FLAG-tagged Tor1 and Tor2 strains were made previously [23]. To tag genomic *mip1*^+^, *tco89*^+^, *ste20*^+^, *sin1*^+^ and *bit61*^+^ with the sequence encoding GFP at the carboxy-terminus, these open reading frames were amplified by PCR and cloned into the plasmids pHYG (*mip1*^+^, *ste20*^+^ and *sin1*^+^) and pNAT (*tco89*^+^ and *bit61*^+^), which contain GFP epitopes and drug markers (hygromycin and clonNAT, respectively). The resulting plasmids were linearized, chromosomally integrated at the endogenous loci and the resulting integrants were verified by PCR sequencing. Genetic crossing was done to make strains that contained GFP-tagged regulatory subunit genes and FLAG-tagged tor1 or tor2 catalytic subunit genes. In the immunoprecipitation experiment, strains were lysed in the extraction buffer (25 mM HEPES–KOH pH 7.5, 200 mM NaCl, 10% glycerol, 0.1% NP-40, 1 mM phenylmethylsulphonyl fluoride, PMSF) supplemented with protease inhibitor cocktail (Sigma).

### 5.4. Strain construction to assay kinase activity in wild-type and mutant target of rapamycin complexes

To isolate Tor1- and Tor2-containing TORC for the kinase assay, we constructed two strains in which the 3FLAG epitope was chromosomally tagged at the amino termini of the *tor2-287* [23] or *tor1-D* mutant gene that was expressed under the native promoter.

### 5.5. The gene disruption of *fkh1*^+^ gene

For disruption of the *fkh1*^+^ gene, one-step gene replacement was used. 5′-upstream (approx. 500 bp) and 3′-downstream (approx. 320 bp) fragments of the *S. pombe fkh1*^+^ gene were amplified by PCR. The fragments were ligated to flank the clonNAT drug-resistant gene, and the resulting DNA fragment was chromosomally integrated onto the *S. pombe* haploid *h*^+^ 972 wild-type to replace the wild-type *fkh1*^+^ gene. Gene deletion was verified by PCR.

### 5.6. Isolation of the strain carrying the *cut1-206* mutant gene tagged with GFP

GFP was chromosomally tagged at the carboxy-terminus of the wild-type and mutant *cut1-20*6 gene. The GFP fragment used for integration carried the kanamycin-resistant gene *kan*^*R*^^+^ and was integrated onto the endogenous wild-type and mutant *cut1-206*. Correct integration was verified by PCR. The resulting chromosomally integrated Cut1–GFP and Cut1-206-GFP were expressed under the native promoter.

### 5.7. Target of rapamycin complexes kinase assay

Growing cells (1 × 10^7^ ml^−1^) of FLAG-tagged strains were lysed in extraction buffer (25 mM HEPES–KOH pH 7.5, 200 mM NaCl, 10% glycerol, 0.1% NP-40, 1 mM PMSF) supplemented with protease inhibitor cocktail (Sigma). Extracts were centrifuged twice (20 min at 7600 r.p.m. and 30 min at 20 000 r.p.m.), and supernatants (160 or 400 mg of total protein) were incubated with anti-FLAG M2 affinity gel (Sigma) for 2 h. Because our data showed that the levels of FLAG–tor2 was more abundant than those of others in the same amount of total cell extract, 160 mg of total cell extract for FLAG–tor2 and 400 mg for the other five proteins were used for incubation with anti-FLAG M2 affinity gel in order to prepare the same amount of immunoprecipitated TOR proteins. The beads were then washed with the extraction buffer. Eluates were obtained by incubation with 150 µg ml^−1^ 3x FLAG peptide (Sigma). To assay the kinase activities, K-LISA mTOR Activity kit was used according to manufacturer instructions (Merck). Mammalian recombinant p70S6K-GST fusion protein was used as the substrate. Phosphorylated S6KT389 was assayed by enzyme-linked immunosorbent assay (ELISA).

### 5.8. Microscopy

DAPI staining was performed as described previously [72]. Cells were fixed with 2.5 per cent glutaraldehyde for 20 min on ice, washed three times with phosphate-buffered saline (PBS) and observed under a fluorescence microscope after staining with DAPI (25 µg ml^−1^). Alternatively, cells fixed with paraformaldehyde were observed after staining with DAPI (0.5 µg ml^−1^), anti-TAT1 (tubulin) antibody and Rhodamin-conjugated phalloidin (actin, 0.165 µM). A BZ9000 microscope (Keyence, Japan) was used.
